# Supplementary figures and images for: JUN mRNA translation regulation is mediated by multiple 5’ UTR and start codon features
Source: PLoS One. 2024 Mar 14;19(3):e0299779. doi: 10.1371/journal.pone.0299779 (PMC10939236; doi:10.1371/journal.pone.0299779)

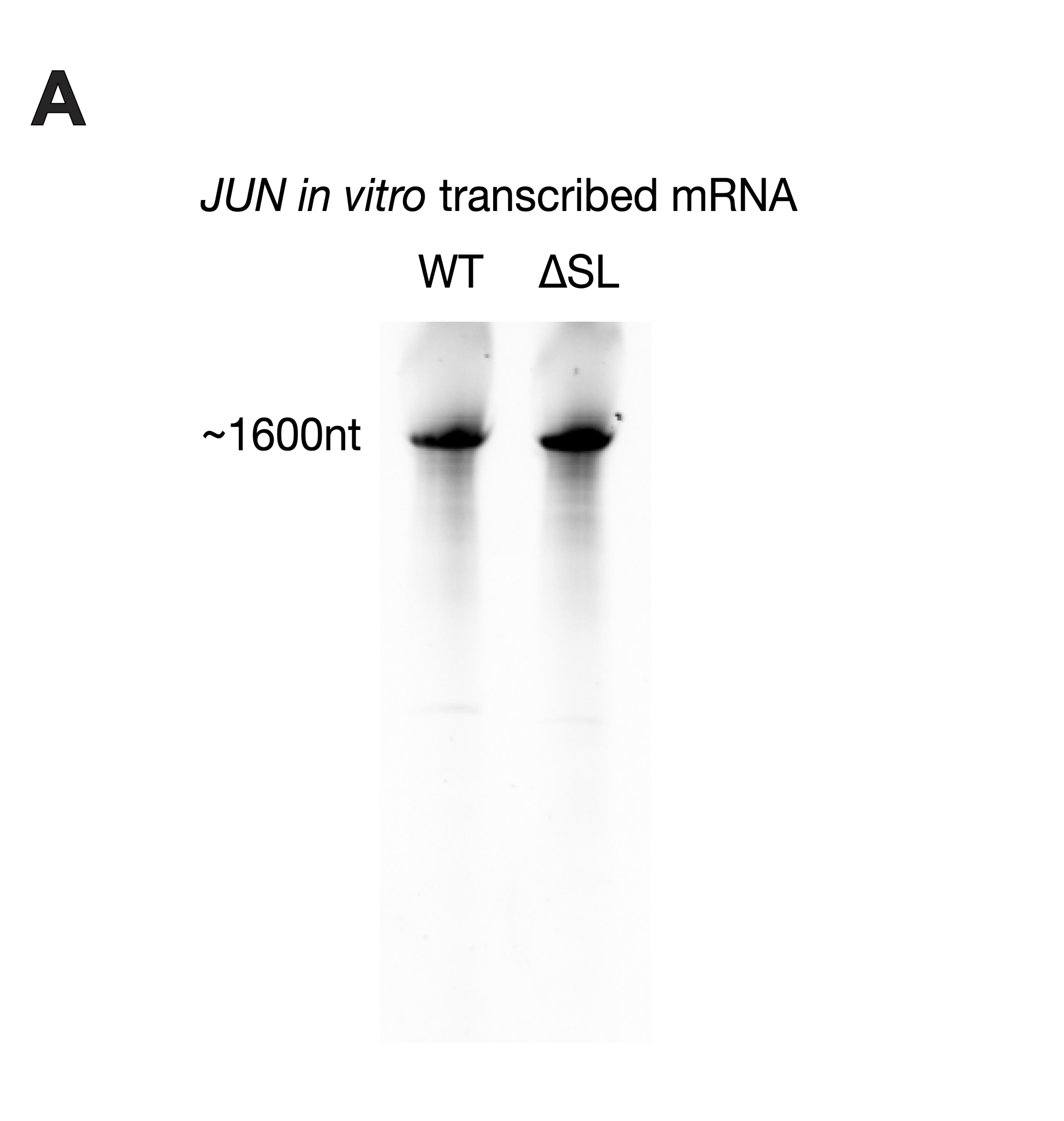

Supplement: S1 Fig — 6% TBE-Urea gel for in vitro transcribed mRNA for the WT or ΔSL JUN 5′ UTR and Nluc CDS reporter constructs. nt, nucleotide. (TIF) [file pone.0299779.s001.tif]

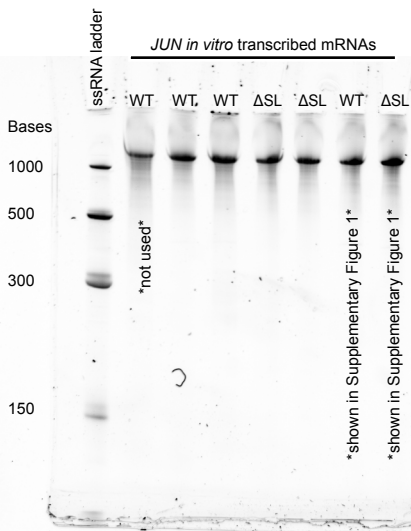

Supplement: S1 Raw image — (PDF) [file pone.0299779.s005.pdf]
