# Supplementary material for: JUN mRNA translation regulation is mediated by multiple 5’ UTR and start codon features
Source: PLoS One. 2024 Mar 14;19(3):e0299779. doi: 10.1371/journal.pone.0299779 (PMC10939236; doi:10.1371/journal.pone.0299779)
Supplement: Attachment — Submitted filename: renamed_94f67.pdf [file pone.0299779.s006.pdf]

Response to reviewers

Reviewer 1

Significance:
The recruitment of eukaryotic translation initiation factors and, consequently translation initiation, are regulated by secondary and tertiary structures found in the 5'-UTR of mRNA. Human JUN mRNA contains a long 5'-UTR with high degree of secondary structure, which facilitates the recruitment of eIF3 for the translation initiation. This is a progressive extension of previous work conducted by the Cate group (Lee et al, 2015 and 2016) where they show that eIF3 binds a stem loop (SL) in 5'UTR and regulates JUN mRNA translation. In this study, authors, identified i) additional sequences adjacent to the SL in 5'UTR, ii) showed the involvement of eIF4A in the regulation of JUN mRNA translation, and iii) the mechanisms of start codon selection in JUN mRNA. However, there are specific questions and concerns that the authors should address. The detailed comments are as follows.

We thank the reviewer for the careful reading and a thorough analysis of our work and for finding it interesting and significant.

Specific comments:

1. In Fig 1, authors identified several important sequences by mutagenesis, which regulate JUN mRNA translation which are interesting, especially, mutants A, F, and G. i) It will be interesting to know what happens to a double mutant of F with A or G. Do any of mutation A or G overcome the mutation F? ii) In mutant F, do eIF3 still binds to stem loop? iii) Do mutants A and G disrupt the binding of eIF3?

We thank the reviewer for raising these interesting questions. We agree it would be interesting to test the effects of these double mutants, and how they affect eIF3 binding. We think this would be a valuable question to explore in future studies, and now mention this in the Discussion.

2. In the presence of any of the mutations mentioned in Fig 1, does overexpression of eIF4A overcome the translation repression?

This is an interesting question. As noted in the Discussion, RocA inhibits translation of the *JUN* reporter mRNA, even with the SL mutation. This suggests that the eIF4A dependence of *JUN* translation is at least partially independent of the eIF3 binding requirement. We think this question would be interesting to explore in a future study.

3. In Fig 2A, "JUN 5' UTR G" wrongly labelled, it is "JUN 5' UTR E".

Thank you for pointing this out! We've corrected this in the figure.

4. In Fig 2A, since mutant A and G are unreactive by SHAPE, also showed significant increase in JUN mRNA translation, do any of these mutations show resistance to RocA treatment?

We think this would be an interesting question to test in a future study. As noted above, inhibition of *JUN* translation by RocA seems to be independent of the action of eIF3, at least with respect to the SL region. We've modified the Discussion to address this point more clearly.

5. Lee et al. (2016) show that the JUN mRNA translation initiation complex lacks eIF4F components. Here, authors showed that eIF4A plays a role in JUN mRNA translation. Can you please explain this discrepancy?

Yes, this is a surprising result of our studies. While our experiments evaluating the role of eIF4A in *JUN* mRNA translation were done mainly in cells, the experiments presented by Lee et al (2016) were performed using *in vitro* translation reactions and sucrose gradients. In the sucrose gradient experiment, Dr. Lee isolated 48S-like complexes for her analysis. We think this represents a very late stage of translation initiation, and eIF4A may be acting "upstream" of this event. This will be a fascinating question to explore in the future. We've made sure the Discussion captures this idea.

6. In Fig 2C and 2D, results are not matching for DMSO treatment. Why does the ΔSL mutant not exhibit a reduction in the 'in-vitro translation' sample, whereas the CAAC+ΔSL mutant does show a reduction?

Recapitulating the translational repression for the *JUN* 5' UTR ΔSL reporter construct has been challenging *in vitro*. It is possible that the mechanism that regulates translation of a reporter mRNA containing this 5' UTR requires elements predominantly active *in vivo*. We've raised this point in the Discussion.

7. Why are two start codons of JUN mRNA significant, as they are only 4 codons apart.

Thanks for raising this question. Previous studies have shown a role for upstream start codons (uAUGs) that are located in close proximity to the downstream–or main–start codon (mAUG) (Smith et al. 2005; Matsuda and Dreher 2006). In this scenario, leaky scanning of uAUGs in a weak translational context allows for backward oscillation of the ribosome and increases translation of the mAUG. This constitutes an alternative mode of translation for mRNAs with these types of start codons. We hypothesize that this could be the case for the *JUN* mRNA, especially given that both start codons and their translational contexts are conserved. We describe these ideas in the Discussion.

Reviewer 2

This manuscript addresses processes that can contribute to c-Jun mRNA translation. Prior reports has documented that Jun mRNA translation is mediated by its direct engagement with eIF3. This manuscript aims to provide some more details to this translational control process, notably some features of the secondary structure of Jun mRNA, and its potential sensitivity to an anti-cancer drug RocA, which targets eIF4A. These goals are met in this study and the manuscript is clearly written and experimental conclusions are supported by rigorous experiments. There are a few concerns, none major, that should be addressed to enhance rigor and clarity.

We thank the reviewer for their positive feedback on our manuscript.

1. For the translation reporter assays involving the 5'-UTR Jun, do changes in mRNA levels (e.g. stability) factor in the luciferase measurements?

We thank the reviewer for raising this point, as it is an important variable to control in mRNA transfection experiments. mRNA stability, as well as transfection efficiency, are known factors that can affect luciferase measurements in translation reporter assays. To account for this, we performed dual-luciferase assays, in which we co-transfected the corresponding *JUN* 5' UTR with Nluc CDS reporter construct together with a control Hemoglobin Subunit Beta (*HBB*) 5' UTR with Fluc CDS. The *HBB* 5' UTR construct is not an eIF3 target and accounts for basal levels of translation given the mRNA stability and transfection efficiency of the sample. For this, luciferase measurements were taken for each of these constructs independently and the Nluc construct measurements were normalized to the Fluc construct measurements. We've clarified this control in the Materials and Methods.

2. Provide more detail justifying the RocA concentration used the study. How do the RocA treatments for Jun translation compare with bulk translation?

This is an important consideration. The RocA concentration used in our experiments was based on the concentrations previously used by Iwasaki *et al*. (2016) in a ribosome profiling experiment to evaluate mRNAs affected by RocA treatment in HEK293T cells. In both our reporter assays and the ribosome profiling experiments *JUN* mRNA translation was repressed by RocA treatment in the concentration ranges explored by Iwasaki *et al.* that did not inhibit bulk translation.

Reviewer 3

The manuscript by Angelica M. Golzalez-Sanchez and coauthors describes novel findings on the translational control of the JUN mRNA. It is a follow up study on previous work published by the same group in Nature in 2015. This current manuscript investigates the sequence/structure requirements of the JUN 5'-UTR for translational control. It further identifies a contribution of eIF4A for this translational control and the requirement of in-frame upstream AUGs. The regulatory elements which the authors describe for the JUN mRNA are important findings for publication, not only because JUN is an oncogenic transcription factor but also the regulation of the JUN mRNA becomes a prototype for studying other mRNAs regulated by similar factors. The studies are well done and the manuscript written very clearly.

We thank the reviewer for their positive feedback on our studies.

Specific comments

1. The study is based on mRNA transfections with the assumption that the levels of the transfected mRNAs are similar among different constructs. Can the authors declare that this is true?

We thank the reviewer for raising this point. As noted above for a similar question by reviewer #2, transfection efficiency is a known factor that can affect luciferase measurements in translation reporter

assays. To account for this, we performed dual-luciferase assays, in which we co-transfected the corresponding *JUN* 5' UTR with Nluc CDS reporter construct together with a control Hemoglobin Subunit Beta (*HBB*) 5' UTR with Fluc CDS. The *HBB* 5' UTR construct is not an eIF3 target and accounts for basal levels of translation given the mRNA stability and transfection efficiency of the sample. For this, luciferase measurements were taken for each of these constructs independently and the Nluc construct measurements were normalized to the Fluc construct measurements. We've clarified this control in the Materials and Methods.

2. In the Roc-A experiments can the authors detect the regulation of the endogenous JUN protein levels?

We thank the reviewer for this question. This is yet to be evaluated since all our experiments were based on using mRNA reporter constructs, not the endogenous mRNA. We note that, by ribosome profiling, Iwasaki *et al.* (2016) saw reduction in *JUN* mRNA translation in the RocA concentration ranges we used. We think this would be interesting to explore in future studies.

3. Can the authors comment if the uAUGs may belong in specific structures in the 5'-UTR that can influence translation independently of their function as ATG codons? Although experiments can be done to show this, I find it outside of the scope of this manuscript.

This is an interesting idea. It is possible, but this is yet to be evaluated. Previous studies, such as Xiang et al (2023), have shown that secondary structures downstream of upstream start codons (uAUGs) regulate start codon selection by unwinding under specific cellular conditions in Arabidopsis thaliana. It is possible that a similar mechanism may occur in human cells, by which a secondary structure in the *JUN* mRNA could be mediating a dynamic selection of the uAUG or of the mAUG under specific cellular conditions. We mention these ideas in the Discussion and think they will be interesting to explore in the future.

4. The Introduction and Discussion are very long for a focused manuscript. However, I enjoyed reading them both. Overall, the findings of this manuscript are interesting and should be published.

We thank the reviewer for their comments, positive feedback, and support for publication of our manuscript.
